# Supplementary material for: Venomix: a simple bioinformatic pipeline for identifying and characterizing toxin gene candidates from transcriptomic data
Source: PeerJ. 2018 Jul 31;6:e5361. doi: 10.7717/peerj.5361 (PMC6074769; doi:10.7717/peerj.5361)
Supplement: Supplemental Information 6 [file peerj-06-5361-s006.gz › FinalOutput_1E-6/Cystatin-1_1/finaltree.pdf]

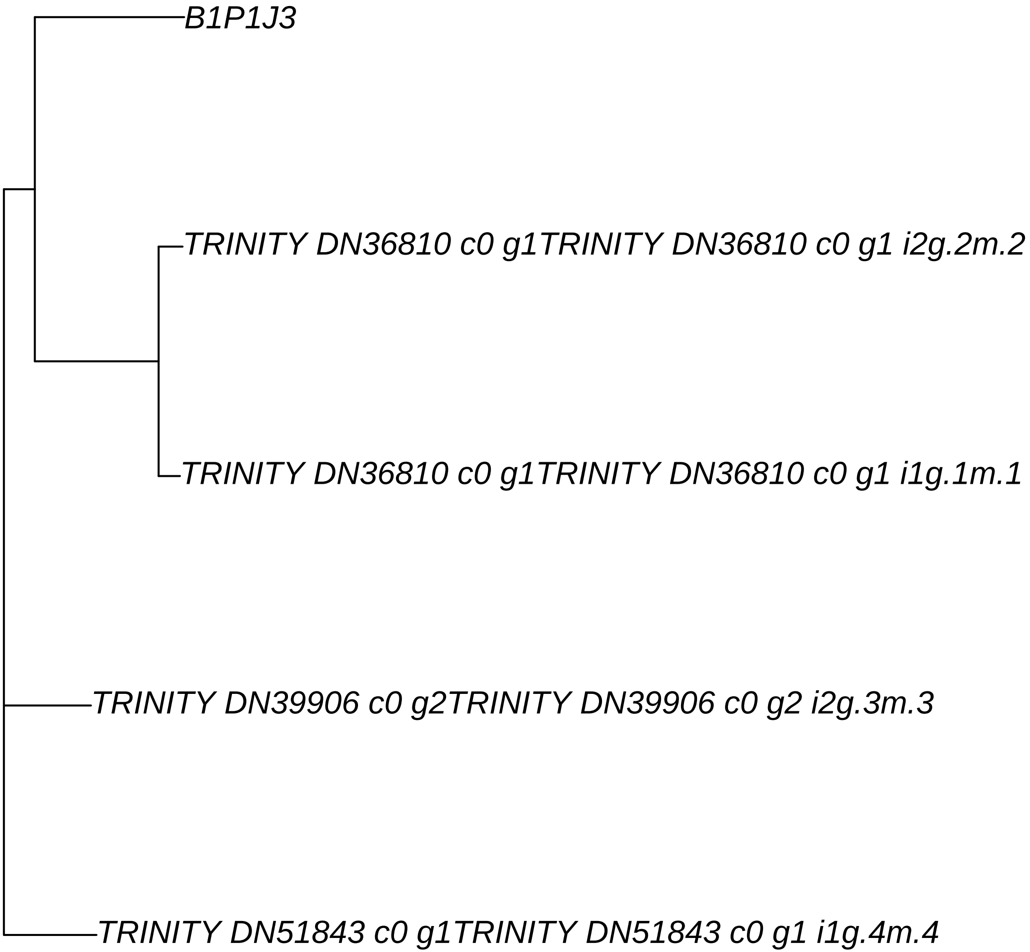
B1P1J3
TRINITY DN36810 c0 g1TRINITY DN36810 c0 g1 i2g.2m.2
TRINITY DN36810 c0 g1TRINITY DN36810 c0 g1 i1g.1m.1
TRINITY DN39906 c0 g2TRINITY DN39906 c0 g2 i2g.3m.3
TRINITY DN51843 c0 g1TRINITY DN51843 c0 g1 i1g.4m.4
